# Robustness in Regulatory Interaction Networks. A Generic Approach with Applications at Different Levels: Physiologic, Metabolic and Genetic

**DOI:** 10.3390/ijms10104437

**Published:** 2009-11-20

**Authors:** Jacques Demongeot, Hedi Ben Amor, Adrien Elena, Pierre Gillois, Mathilde Noual, Sylvain Sené

**Affiliations:** 1 Université J. Fourier de Grenoble, TIMC-IMAG, CNRS UMR 5525, Faculté de Médecine, 38700 La Tronche, France; E-Mails: Hedi.Ben-Amor@imag.fr (H.B.); Adrien.Elena@imag.fr (A.E.); Pierre.Gillois@imag.fr (P.G.); 2 Université de Lyon, École Normale Supérieure Lyon, LIP, CNRS UMR 5668, 69007 Lyon, France; 3 Université d’Evry Val d’Essonne, IBISC, CNRS FRE 3190, 91000 Evry, France; 4 IXXI, Institut rhône-alpin des systèmes complexes, 69007 Lyon, France; E-Mails: Mathilde.Noual@ens-lyon.fr (M.N.); Sylvain.Sene@ibisc.univ-evry.fr (S.S.)

**Keywords:** robustness in regulatory interaction networks, attractors, interaction graph boundary, interaction graph core, critical node, critical edge, updating mode, microRNAs

## Abstract

Regulatory interaction networks are often studied on their dynamical side (existence of attractors, study of their stability). We focus here also on their robustness, that is their ability to offer the same spatiotemporal patterns and to resist to external perturbations such as losses of nodes or edges in the networks interactions architecture, changes in their environmental boundary conditions as well as changes in the update schedule (or updating mode) of the states of their elements (*e.g*., if these elements are genes, their synchronous coexpression mode versus their sequential expression). We define the generic notions of boundary, core, and critical vertex or edge of the underlying interaction graph of the regulatory network, whose disappearance causes dramatic changes in the number and nature of attractors (*e.g*., passage from a bistable behaviour to a unique periodic regime) or in the range of their basins of stability. The dynamic transition of states will be presented in the framework of threshold Boolean automata rules. A panorama of applications at different levels will be given: brain and plant morphogenesis, bulbar cardio-respiratory regulation, glycolytic/oxidative metabolic coupling, and eventually cell cycle and feather morphogenesis genetic control.

“*In nova fert animus mutatas dicere formas corpora... Unus erat toto naturae vultus in orbe, Quem dixere chaos: rudis indigestaque moles, Nec quidquam, nisi pondus iners, congestaque eodem non bene junctarum discordia semina rerum…”. I want to speak about bodies changed into new forms... Nature appeared the same throughout the whole world, What we call chaos: a raw confused mass, Nothing but inert matter, badly combined discordant atoms of things, confused in the one place...*

*(Ovide, Metamorphoses, 1^st^ Book, 10 A.D.).*

## 1. Introduction

**Figure 1.**
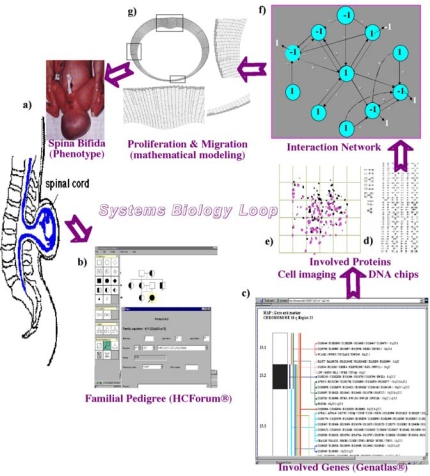
Systems biology circuit with (a) phenotype observation (spina bifida), (b) pedigree inquiry (proving the familiar origin, with affected in black and healthy carriers in bicolor), (c), (d) genomic and proteomic data (karyotypes, databases and DNA chips), (e) cell imaging and concatenation of these elements into a (f) regulatory interaction network and (g) mathematical model allowing the simulation *in silico*.

**Figure 2.**
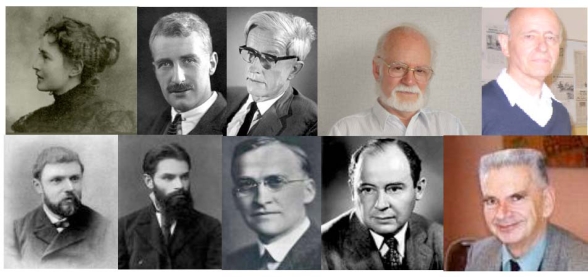
Some contributors to the kinetic equations ruling the regulatory interaction networks dynamics (top from left to right): M. Menten, A. Hill, M. Delbrück, R. Thomas, J. Thiéry; some contributors to the theory of dynamical systems (bottom from left to right): H. Poincaré, A.M. Lyapunov, G.D. Birkhoff, J. von Neumann, R. Thom.

The cell metabolism is regulated by interaction networks [1,2] bringing together elementary macromolecules like genes and their expression products, proteins, in a complex organization made of numerous weak interactions (due to physicochemical forces like electrostatic or van der Waals forces). The aim of this paper is to show how mathematical networks theories like graphs and dynamical systems theories are necessary to give a mechanistic description of how a cell, a tissue or an organ work from the emergent properties of their constitutive interacting metabolisms. Regulatory interaction networks are made of elements (*e.g.*, genes or proteins) in interaction and they control important cell or tissue functions like proliferation and differentiation. Their dynamics depends highly on the relationships and delays between the kinetics of creation and transformation of the networks elements. More generally, a system is a set of elements in interaction and the cell (resp. tissue) organisation is a biological system, considered as a pyramid of components made of interacting macromolecules (resp. cells). Their observed spatiotemporal behaviours (phenotypes) are explained in the framework of Systems Biology, which studies biological systems in a circuit of complexity from data acquisition to reconstruction of regulatory interaction network (inverse problem) allowing direct predictions by modeling and simulating it *in silico* (Figure 1). The complexity of living systems deals with common rules prescribing how macromolecules, cells and tissues are connected into integrated regulatory networks with architectural similarities both inside the cell and between the cellular organizations. Besides, we notice that “network motifs” are crucial to the complexity paradigm. They are common patterns of interconnections between elements as well as super-structures, modeled by deterministic kinetic non-linear rules coming from the classical enzymology and from the genetic networks theory [3–11], *e.g.*, from Henri-Michaelis-Menten, Hill, Monod-Wyman-Changeux and Thomas equations (Figure 2). Probabilistic versions of these rules exist accounting for the variability of the systems trajectories around their average behaviour [12,13]. The mathematical tools from the theories of dynamical systems [14–20] come from numerous contributors from H. Poincaré to R. Thom (Figure 2).

**Figure 3.**
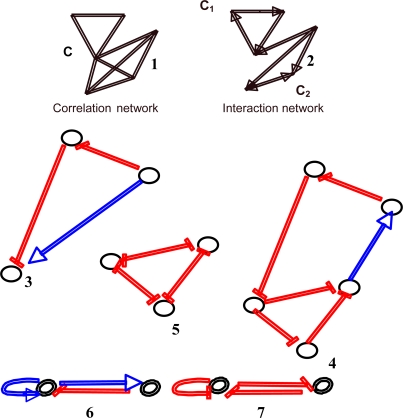
Example of some motifs observed in regulatory interaction networks (inhibitions are in red and activations in blue): non-directed correlation (1) and directed interaction (2) network, coherent feed-forward double path (3), triple negative and quintuple positive circuits (4), 3-switch (5), negative (6) and positive regulons (7).

The interaction graphs associated to the regulatory interaction networks are inferred from the experimental data and from the literature [21–23]. For instance, gene co-expression data may be used for building correlation networks from directional correlations or logical considerations about the observed fixed configurations and then completing the graphs obtained this way by orienting, signing and valuating their edges, hence creating new connected components (like C_1_ and C_2_, from C, on Figure 3 top). Interaction graphs architecture contains simple motifs (as those from Alon’s work in genetic networks [24] and from metabolic and physiologic networks [25,26]) with two to five nodes. Some examples of motifs are given in Figure 3 and systematically studied in [27]. Among these motifs, we will use, in the following, the negative (respectively positive) circuits, *i.e.*, closed paths between nodes having an odd (respectively even) number of inhibitions like in the triple negative and quintuple positive circuits, in the 3-switch (three nodes fully connected with only inhibitions between them) and in the negative and positive regulons, the most simple motifs having both one positive and one negative circuit. When there is no circuit inside the motifs, we call coherent (resp. incoherent) feed-forward double path a couple of paths with same initial and final nodes, and same (resp. different) global signs.

## 2. Preliminary: Notations and Definitions

### 2.1. Definition of an Attractor and of Its Basin

The definitions of attractors are numerous. We have chosen here a version available both for continuous and discrete cases and the only consistent with all encountered practical situations [28,29]. We define first the *Birkhoff limit set L(x)* of an initial condition *x* in the state space *E* as the set of accumulation points of a trajectory *T*, where *T(x,s)* is the state reached at time *s* from the initial condition *x* and *B(y,ε)* is the set of states at distance of *y* less than *ε* (reduced to {*y*} in the discrete case):

L(x)={y∈E;  ∀ɛ>0,  ∀t∈R,  ∃s>t/B(y,ɛ)∩T(x,s)≠ø}

The *stability basin* of a subset *A* of *E* is the set of initial conditions *x* not in *A*, but such as *L(x) ⊂ A*. Let us denote by *Ā* the set *A* completed by possible shadow trajectories [30]. An *attractor A* verifies:

- *A* is a fixed set for the composed set operator *LoB: A = L(B(A))*,
- there is no set *C ⊃ Ā, C ≠ Ā*, verifying i),
- there is no *D ⊂ A*, *D ≠ A*, verifying (i) and (ii).

**Figure 4.**
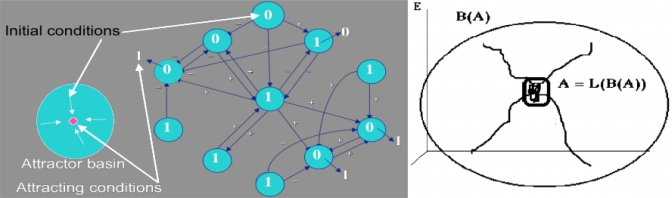
Definition of an attractor and its basin in the case of a Boolean regulatory network; the initial condition is indicated in blue disks and the final attracting conditions for the majority rule after synchronous iterations (the state of a node is 1 if activating neighbours number is equal to or more than those of inhibiting ones) are given outside the disks (left); attractor *A* and its basin *B(A)* (right).

An attractor *A* is invariant in the dual operations consisting firstly in considering all the trajectories of its basin (from all initial conditions not in *A*, but finishing their life in *A*) and secondly to restrict them to their ends of life (Figure 4).

**Figure 5.**
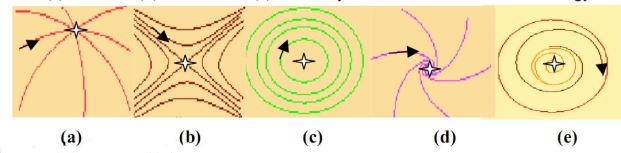
Different categories of attractors: trajectories landscapes for (a) a node, (b) a saddle, (c) a centre, (d) a focus and (e) a limit cycle, in the Poincaré’s terminology.

The simplest case of attractors is the fixed point called node [14–17], to which converge all trajectories of its stability basin (Figure 5); among the other fixed points, the focus is also an attractor, but neither the centre nor the saddle. The attractors fixed points can bifurcate toward a limit cycle when the parameters values of the dynamical system change (Figure 5). The epigenetic differences including those involved in cell differentiation can be understood in the framework of the regulatory networks in terms of existence of multiple attractors [8], and the presence of a positive circuit in their interaction graph is a necessary condition for observing multiple fixed points. The second part of the sentence has been conjectured in [31] and proved both in continuous [32–37] and discrete cases [38–40], where attractors depend in general on the updating mode of the network: sequential if each node is updated in a deterministic or random ergodic order, parallel if all nodes are updated synchronously and block-sequential if the synchrony is available only into blocks updated sequentially.

### 2.2. Degree, Connectivity and Connectedness

#### 2.2.1. Undirected Graph

An *undirected graph G* is a pair *(V,E)*, where *V* is a set whose elements are called *vertices* (or *nodes*), and *E* is a set of non ordered pairs of vertices, called *edges*. The vertices of an edge are called its *endvertices*. Two vertices of an undirected graph belonging to the same edge are called *incident* with this edge. Any edge is called *incident* with its endvertices. Two edges (resp. vertices) are *adjacent* if they are incident with one common endvertex (resp. edge). A *path* (or *m-path*) is an ordered sequence of *m* edges in which two consecutive edges are adjacent, by sharing one endvertex not incident with any previous edge of the sequence, except possibly, for the last edge, with the first edge. In this case, the path is called a *cycle* (or *m-cycle)*. In an undirected graph *G*, two vertices *u* and *v* are called *connected*, if *G* contains a path from *u* to *v*. Otherwise, they are called *disconnected*. A graph is called *connected* if every pair of distinct vertices in the graph can be connected through some path. A *connected component* is a maximal connected subgraph of *G*. Each vertex belongs to exactly one connected component, as does each edge. If *u* and *v* are vertices of *G*, then a collection of paths between *u* and *v* is called *vertex-independent* if no two of them share a vertex (other than *u* and *v* themselves). Similarly, the collection is *edge-independent* if no two paths in it share an edge. The greatest number of vertex-independent paths between *u* and *v* is called the *local vertex-connectivity κ(u,v)* and the greatest number of edge-independent paths between *u* and *v*, the *local edge-connectivity λ(u,v)*. G is called *r-vertex-connected* (resp. *r-edge-connected*) or has a *vertex-connectivity κ(G)* (or an *edge-connectivity λ(G)*) equal to *r*, if each pair of vertices of *G* has the same vertex-connectivity (resp. edge-connectivity) *r*. The *degree* (or *valency*) of a vertex is the number of edges that connect to it, where an edge that connects to the vertex at both ends, a 1-cycle, called also *loop*, is counted twice. The *average degree* of *G* is equal to *d* = 2|*E*|/|*V*|, where |*E* |(resp. |*V*|) denotes the number of edges (resp. vertices) of the graph. The vertex-connectivity of a graph is less than or equal to its edge-connectivity: *κ(G) ≤ λ(G)*. Both are less than or equal to the *minimum degree* of *G*, equal to the minimum value of the degree of its vertices.

#### 2.2.2. Regular Graph

A *regular graph* is a graph where each vertex has the same number of neighbours, *i.e.*, every vertex has the same degree. A regular graph with vertices of degree *r* is called a *r*-*regular graph*. For a random *r*-regular graph *G* (chosen uniformly in the set of *r*-regular graphs), the following result holds, relating degree and edge-connectivity [55]: assume 3 ≤ *r* ≤ *c_0_n*, for some small positive constant *c_0_*, then with probability tending to 1 as *n* tends to infinity, *G* is *r*-edge-connected.

#### 2.2.3. Weighted and Signed Graph

A graph is a *weighted graph* if a real number, called *weight*, is assigned to each edge. Such weights represent in regulatory networks the intensity of the influence (repression/inhibition or induction/activation) of an element on the others, depending on the problem. The *weight of the graph* is the sum of weights of all edges. A *signed graph* is a weighted graph with weights valued in {−1,0,1}.

#### 2.2.4. Directed Graph

**Figure 6.**
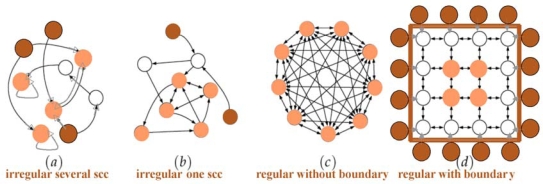
From realistic biological regulatory networks (a & b) to regular networks (c & d) with a reduction of the strongly connected components (scc) from 3 to 1; the boundary (respectively core) of the networks is indicated in dark (respectively light) brown nodes.

A *directed graph* or *digraph G* is a pair (*V,E*), where *V* is the set of vertices, and *E* is a set of ordered pairs of vertices, called *directed* or *oriented edges*. In a digraph *G*, a path is oriented. Thus, the concept of *circuit* replaces that of cycle. A digraph *G* is *connected* if the undirected underlying graph obtained by replacing all directed edges of *G* with undirected edges is a connected graph. A digraph is *strongly connected* if it contains a directed path from *u* to *v* and a directed path from *v* to *u*, for every pair of vertices (*u,v*). The *strongly connected components (scc)* are the maximal strongly connected subgraphs. These strongly connected components (scc) are less numerous in the oriented graphs than in their non oriented versions like in the correlation graphs (Figure 3). Passing from regulatory networks of biology to regular networks of physics (*e.g.*, Ising model graph), we have a drastic reduction of the scc’s (Figure 6).

#### 2.2.5. Indegree and Outdegree

For a vertex of a digraph, the number of head endvertices (resp. tail endvertices) of edges adjacent to this vertex is called the *indegree* (resp. *outdegree*) of the vertex. The indegree (resp. outdegree) is the number of incoming (resp. outcoming) edges in a vertex *v* and is denoted *deg*^−^(*v*) (resp. *deg^+^*(*v*)). A vertex *v* with *deg*^−^*(v) = 0* is called a *source*, as it is the origin of each of its incident edges. Similarly, a vertex *v* with *deg^+^(v) = 0* is called a *sink*. The degree sum formula states: *∑_v∈V_deg^+^(v) = ∑_v∈V_deg^−^(v) =* |*E*|. Hence the average indegree is equal to the average outdegree.

#### 2.2.6. Connectedness and Connectivity in Graphs

The *connectedness* (or *connectance*) [56] in a directed graph is equal to *C =* |*E*|/|*V*|*^2^* and, in an undirected graph without loops, to *C =* 2|*E*|/[|*V*| *(*|*V*| *-* 1*)]*. Gardner and Ashby [4] have studied the relation between the probability of stability and the connectedness of large linear dynamical systems observed in biology [57,58]. We can notice that the *connectivity* in the Kauffman’s sense [41] is the average indegree, which is also equal in directed graphs to *c = C*|*V*|; *c* is set to a constant, however, it is possible to let it be random, chosen under various distributions [50,51], with *average connectivity c*.

### 2.3. Kauffman Boolean Networks

Let us denote by *R* a genetic regulatory network whose number of genes equals *n*. This network, whose *size* is defined by *n,* is modeled by a graph of *n* vertices representing the *n* genes. Each gene has two possible activity states (expression and silence) such that the state *x_i_(t)* of the gene *i* equals *0* (resp. *1*) if the gene is inactive (resp. active) at time *t*. We denote the whole configuration of the network at time *t* by: *x(t) = (x_i_(t))_i∈R_; x(t)∈ Ω =* {0,1}*^n^*, where *Ω* is the set of all possible Boolean configurations on *R*. A *Kauffman Boolean network* consists of *n* interacting elements whose states *x_i_(t)* (i from 1 to *n*) are binary variables [41–51]. Each gene *i* is connected to *k* other genes *(i_1_,...,i_k_)* and its state is updated according to a specific rule *x_i_(t+*1*) = F_i_[x_i1_(t),..., x_ik_(t)]*, where *F_i_* is a Boolean function, and the *x_ij_(t)* are the states of the units connected to *i*, which may or may not include *i* itself. The Boolean function *F_i_* is represented by a truth table that lists its outputs for each set of inputs values. For each *F_i_* with *k* variables, there are *K =* 2*^k^* possible sets of inputs values, yielding 2*^K^* different possible functions.

### 2.4. Threshold Boolean Automata Networks

A threshold Boolean automata network with *n* genes can be represented by an oriented graph in which the *n* vertices correspond to genes and directed edges to the interactions between these genes. Each interaction from gene *j* to gene *i* is characterised by an interaction weight *w_ij_* which gives the intensity of the influence that gene *j* has on gene *j* and, consequently, of the role played by gene *j* through the protein it expresses, which can repress or induce the expression of gene *i*. Being given an arbitrary regulatory network, we associate to it an interaction matrix *W* of order *n*-*n*, whose general coefficient *w*_ij_ corresponds to the interaction weight with which gene *j* acts on gene *i*. More precisely, the coefficient *w_ij_* can be positive or negative depending on the fact that gene *j* tends to respectively activate or inhibit gene *i*, and is null if *j* has no influence on *i*. Let *V_i_* be the *neighbourhood* of gene *i*, *i.e.*, the set of genes *j* having an influence on *i* (*j∈V_i_ ⇔ w_ij_ ≠*0) and *H* the *interaction potential*:

(1) $$H(x_{i}(t))=\sum_{j\in Vi}w_{ij}x_{j}(t)-\theta_{i}$$

where *x*(*t*) *∈ Ω* = {0,1}*^n^* and *θ_i_* is the *i^th^ activation threshold*, *i.e.*, the interaction potential to be overtaken for activating *i*. The threshold Boolean automata rule [52–54] is:

(2) $$x_{i}(t+1)=G(H(x_{i}(t)))$$

where *G* is the Heaviside (or *sign-step*) function: *G(y) =* 0, if *y ≤* 0 and 1 otherwise. In certain applications, the state *0* can be replaced by the state *−1*; in this case, *G(y) =* −1, if *y ≤* 0 and 1 otherwise.

### 2.5. Attractors in Kauffman Boolean Networks and Threshold Boolean Automata Networks

In a random Kauffman Boolean network of size *n* with connectivity *c* (*i.e.*, with an average indegree equal to *c*), we choose randomly among the 2*^K^*, where *K =* 2*^c^* possible random Boolean functions determined by the *c* (on average) neighbours of any node. For *c =* 1/2 and for the Boolean functions identity and negation chosen with the same probability, there are *N = O(√(n))* states belonging to the attractors [41–45] and the number of possible limit cycles of length *L* has been recently proven to be in general exponential according to *L* [44–49]. In a random threshold Boolean automata network, with *c =*2 (*i.e.*, with on average 2 neighbours acting on the same gene with weights randomly chosen at values 1 or −1), we conjecture that the number *N* of attractors in the sequential updating mode (each vertex being sequentially updated following a deterministic or random ergodic order) verifies [53,54]:

$$2^{S}\leq N\leq 2^{S^{\prime }},$$

where *S* is the number of the strongly connected components with a positive circuit and *S’* the number of the positive circuits (counted only 1 time if connected).

## 3. Notions of Boundary, Core, Critical Node and Critical Edge of a Regulatory Interaction Network

### 3.1. Boundary and Core

The notions of boundary and core of a regulatory interaction network come from graph theory. The notions of critical node and edge are more specific in biological applications. For defining correctly these notions, we consider a threshold Boolean automata network: its *core* is defined by the set of nodes *k* whose *eccentricity e*(*k*) (*i.e.*, the maximal length of the shortest paths to reach from node *k* all the others) is minimal and the boundary by the set of *sources*, *i.e.*, nodes whose indegree is null (Figure 6). The *diameter* of a graph is equal to the maximum of the eccentricities of its vertices.

### 3.2. Critical Node and Critical Edge

An edge in a regulatory network is *critical* if its suppression causes the loss of at least one attractor.

**Figure 7.**
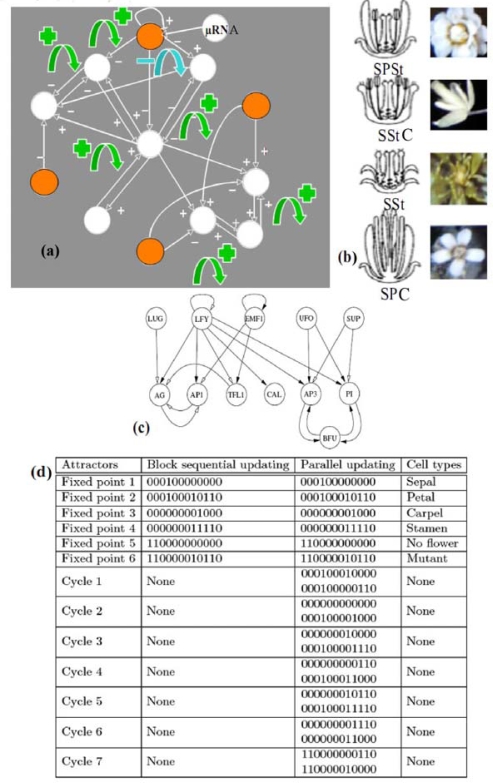
(a) Regulatory network controlling the flowering of *Arabidopsis thaliana* [52]. Sources are coloured in orange, a micro-RNA acting on one of them. Green (resp. blue) arrows indicate the location of the positive (resp. negative) circuits. (b) Phenotypic expressions with some of the different possible attractors, with the symbols Sepals (S), Petals (P), Stamens (St) and Carpels (C): SPStC, SStC, SSt, SPC. (c) Simplified network with conservation of attractors. (d) Attractors, with genes in the following order: EMF1, TFL1, LFY, AP1, CAL, LUG, UFO, BFU, AG, AP3, PI, SUP.

It is the case in the example of the flowering regulatory network of *Arabidopsis thaliana* (cf. Figure 7(a) and [23,59]). A simplified version [Figure 7(c)] of the primitive interaction graph keeping the same attractors, has eight boundary nodes (of eccentricity 2), two strong connected components in its core, four positive and one negative not connected circuits. It has six fixed points and seven limit cycles of order 2 in the parallel (co-expressed) updating mode (which is a consequence of a theorem of [60]) and respects the inequality conjectured in Section 2, because the number *N =* 6 of attractors in the sequential updating mode verifies: 2*^2^ ≤ N ≤* 2*^4^*.

The flowering regulatory network of Arabidopsis thaliana is sensitive to the change of updating mode, because it loses all the seven limit cycles observed in the parallel mode when we let it evolve with the sequential mode. The flowering network is also sensitive to the knockout of the gene EMF1 by a micro-RNA, which causes the loss of two fixed points among six [61–63].

## 4. Theoretical Complements

### 4.1. Potential Regulatory Networks

A continuous potential differential equation on R*^n^* is defined by:

(3) $$\forall i=1,\ldots ,n,\;dx_{i}/dt=-\partial P/\partial x_{i}$$

where the *potential P* is a real continuously differentiable function on R*^n^*. In the same way, a *potential regulatory network* on the discrete state space *E* is defined by [64,65]:

(4) $$x_{i}(t+1)=h(-\Delta P/\Delta x_{i}+x_{i}(t))$$

where *P* is a real function (*e.g.*, a polynomial with real coefficients) on *E* and *h* a function from R to *E*, with boundary conditions ensuring that the flow remains in *E*. For example, in the Boolean case, we choose for *h* the Heaviside function *G*. In the integer case (*E ⊂ Z^n^*), *h* is the identity, *P* a polynomial with integer coefficients and ∀*i =* 1*,...,n*, *Δx_i_ ∈* {−1,0,1}. Then (5) is the discrete equivalent of (3):

(5) $$\Delta x_{i}/\Delta t=h(-\Delta P/\Delta x_{i}+x_{i}(t))-x_{i}(t)$$

***Example:*** in the Boolean case, if *P(x) = ∑_k_(^t^txA_k_x)x_k_ + ^t^xWx+Θx*, where *A = (a_ijk_)* is an interaction tensor, with *A_k_ = (a_ij_)_k_* as marginal matrices and *a_iii_* = 0, *W = (w_ij_)* an interaction matrix and *Θ = (θ_i_)* a threshold vector, we have for the partial derivatives of *P*:

(6) $$\Delta P/\Delta x_{i}=\sum_{j,k}(a_{ijk}+a_{jik}+a_{jki})x_{j}x_{k}+\sum_{j}(w_{ij}+w_{ji})x_{j}+\theta_{i}+[w_{ii}+\sum_{j\neq i}(a_{ijj}+a_{jij}+a_{jji})\;x_{j}]\Delta x_{i}$$

Then the potential regulatory network associated to *P* is defined by:

$$\Delta x_{i}/\Delta t=\Delta x_{i}=-\Delta P/\Delta x_{i}=-\sum_{j,k}(a_{ijk}+a_{jik}+a_{jki})x_{j}x_{k}-\sum_{j}(w_{ij}+w_{ji})x_{j}-\theta_{i}-[w_{ii}+\sum_{j\neq i}(a_{ijj}+a_{jij}+a_{jji})\;x_{j}]\Delta x_{i}$$

Hence we have: Δ*x_i_* = −[*∑_j,k_*(*a_ijk_* + *a_jik_* +*a_jki_*)*x_j_(t)x_k_(t)*+*∑_j_(w_ij_* + *w_ji_)x_j_(t) + θ_i_]*/[1+*w_ii_* + *∑_j ≠i_*(*a_ijj_* +*a_jij_*+*a_jji_*)*x_j_(t)*] and *x_i_(t + 1)* = *G(Δx_i_ + x_i_(t))* = *G(-ΔP/Δx_i_ +x_i_(t))*, where G is the Heaviside function. From (6) we derive:

(7) $$x_{i}(t+1)=G(-[\sum_{j,k}(a_{ijk}+a_{jik}+a_{jki})x_{j}(t)x_{k}(t)+\sum_{j}(w_{ij}+w_{ji})x_{j}(t)+\theta_{i}]/[1+w_{ii}+\sum_{j\neq i}(a_{ijj}+a_{jij}+a_{jji})x_{j}(t)]+x_{i}(t))$$

**Figure 8.**
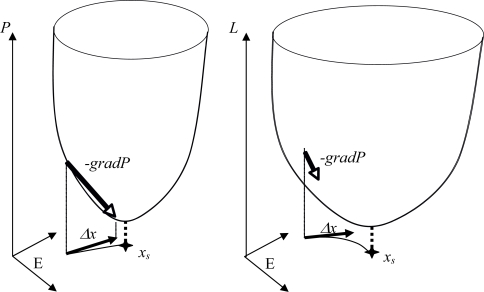
Potential networks with Δ*x = -gradP* (left) and with a Lyapunov function *L* decreasing on its trajectories (right). The potential network has a fixed point *x_s_* at the projection of the *P* sink and presents an identity between the discrete velocity *Δx/Δt* and the opposite of the gradient of *P* (left); on the contrary, there is only identity between the projection of the *P* sink and the fixed point localization in the case of a Lyapunov function *L* decreasing along the trajectories (right).

In the Boolean case, let suppose that *A* = 0, *P(x) = ^t^xWx + Bx*, with *w_ii_* = 1, and each sub-matrix on any subset *J* of indices in {1*,...,n*} of *W* is non positive. Then *P* decreases on the trajectories of the potential automaton defined by *x_i_(t +* 1*) = G(-ΔP/Δx_i_ + x_i_(t))* for any mode of implementation of the dynamics (sequential, block sequential and parallel). This network is a threshold Boolean automata neural network whose stable fixed configurations (Figure 8) correspond to the minima of *P* [64].

### 4.2. Hamiltonian Networks

A *Hamiltonian energy* function *H* can be defined in a regulatory network of size *n*, from a kinetic energy term minus a potential energy term, both energies becoming constant at the dynamic equilibrium. The definition of *H* requires the new following variables:

$$Y_{i}(t)=\Delta x_{i}/\Delta t=[x_{i}(t+1)-x_{i}(t)]/(t+1-t)=x_{i}(t+1)-x_{i}(t)\;\text{and}\;T_{i}(t)=[x_{i}(t+1)+x_{i}(t)]/2$$

*Y_i_(t)* can be interpreted as the rate at which the system changes its state *x_i_(t)* at node *i* and time *t* and *T_i_(t)* as the mean local state over the time interval [*t,t* + 1]. Then let us define the Hamiltonian energy *H* equal to the *kinetic energy: E_c_(t) = ∑_i=0,...,n-1_ Y_i_(t)^2^/2*, to which we substract the *potential energy* defined by: *ΔE_p_(t) = ∑_i=0,...,n-1_ Z_i_(t)ΔT_i_*, where Z_i_(t) = ΔY_i_/Δt =Y_i_(t + 1)-Y_i_(t) = x_i_(t + 2)-2x_i_(t + 1) + x_i_(t) plays the role of an acceleration. We have:

(8) $$H(t)=E_{c}(t)-E_{p}(t)$$

Hence: *ΔH(t) = E_c_(t+1)-E_c_(t)+E_p_(t)-E_p_(t+1) = ∑_i=0,...,n-_*_1_[Y_i_(t+1)^2^-Y_i_(t)^2^)]/2-*∑_i=0,...,n-1_Z_i_(t)ΔT_i_ = ∑_i=0,...,n-1_[(Y_i_(t+1)+Y_i_(t))(Y_i_(t+1)-Y_i_(t))/2-Z_i_(t)ΔT_i_] = ∑_i=0,...,n-1_[(T_i_(t+1)-T_i_(t))ΔY _i-_ Z_i_(t)ΔT_i_] = 0*, and we can write:

(9) $$\Delta H/\Delta Y_{i}=\Delta T_{i}/\Delta t\;\text{and}\;\Delta H/\Delta T_{i}=-\Delta Y_{i}/\Delta t$$

A discrete system verifying the equations (9) for a Hamiltonian energy function *H* is called *Hamiltonian* and is *conservative* for *H* [66], because it keeps *H* constant.

Let us consider now a *r*-regular network with only identity and negation functions. In such a network, a positive (resp. negative) circuit is conservative for the average of the kinetic part of the Hamiltonian energy *H* because *E_c_(t + n) = E_c_(t)* (resp. *E_c_(t + 2n) = E_c_(t)*), and hence, the quantity:

$$<E_{c}{>}_{n}(t)=\sum_{i=0,\ldots ,n-1}\;E_{c}(t+i)/n\;(\text{resp}.\;<E_{c}{>}_{2n}(t)=\sum_{i=0,\ldots ,2n-1}E_{c}(t+i)/2n),$$

we call *average kinetic energy* over the time interval [*t*,*t* + *n-*1] (resp. [*t*,*t* + 2*n-*1]), is constant (which is not the case in general before the system reaches its attracting behaviour). As direct consequence, all trajectories on the circuits are fixed configurations or limit cycles with constant average kinetic energy.

### 4.3. Relationships between Kauffman Boolean and Threshold Boolean Automata Networks

A Kauffman Boolean network with only identity and negation functions [45,67], as well as states *x(t)∈*{0,1}*^n^*, can be considered as a threshold Boolean automata network with states *y(t)∈*{−1,1}*^n^* by considering variables *y_i_(t)* = 2*x_i_(t)-* 1, where *x_i_(t)∈*{0,1}. It suffices to define thresholds as 0 and weights as follows: for identity function, *w_ii+1_* > *w_ii_* > 0 and for the negation function, -*w_ii+1_* > *w_ii_* > 0.

Reciprocally, each threshold Boolean automata network, because its rule corresponds to a Boolean function from Ω = {0,1} *^n^* to {0,1}, can be expressed in terms of Kauffman Boolean networks.

### 4.4. Relationships between Undirected and Directed Graphs

Lemma 1 proves a relationship existing between the number of non-oriented edges defined on an undirected graph *G* and the mean number of directed edges chosen on the directed versions of *G*.

**Lemma 1:** for any undirected graph *G* having *m* non oriented edges, the mean number of oriented edges we can define on *G* from the non oriented configuration is equal to *4m/3*.

**Proof:** Let us note *<O>* the mean number of oriented edges we can construct from a configuration of m non oriented edges; then, if exactly k from the *m* non oriented edges are decomposed into two oriented opposite connections, we have *C_m_^m-k^2^m-k^* different ways to dispatch the not double connections into the *(m-k)* other non oriented edges; we can write:

(10) $$<O>=\sum_{k=0}^{m}(2k+m-k)C_{m}^{m-k}2^{m-k}/\sum_{k=0}^{m}C_{m}^{m-k}2^{m-k}=4m/3$$

From Lemma 1, the average indegree *c* (or connectivity in Kauffman’s sense) of the directed graphs we can randomly construct from their undirected version of degree *d* is such as *c=4m/3n*, where *m*=*nd*/*2*; then *2nd/3*=*nc* and a directed average indegree *c* corresponds to an undirected degree *d=3c/2*.

### 4.5. Circuits

Let us define now *X_r_*, the number of non-oriented circuits of length *r* in a random undirected *d*-regular graph of order *n*. In [68–70], it is shown that the random variables *X_r_*, for 3 *≤ r ≤ g*, are asymptotically distributed as independent Poisson variables with means equal to *(d-*1*)^r^/*2*r=(*3*c–*2*)^r^/r*2*^r+1^* (2*^r^*^−1^/*r*, if *c*=2), with *d* = *d*(*n*) and *g* = *g*(*n*) allowed to increase with *n*, provided that: *(d-*1*)^2g–1^* = *o*(*n*), hence we have:

$$g=[{\text{Log}}_{2}[o(n)]+1]/2,\;\text{if}\;c=2,\;\text{which}\;\text{holds}\;\text{for}\;g\leq 5,\;\text{if}\;n=22.10^{3}\approx 2^{14.5}\;\text{and}\;o(n)=n^{2/3}$$

There are *C^nd/2^_n(n-1)/2_* random undirected *d*-regular graphs of order *n*, each bringing on average 2*^r^*^−1^/*r* non-oriented circuits of length *r*, if the average in-degree *c* = 2, and each undirected edge of a non-oriented circuit of length *r* gives birth to 1 (counted twice in case of a loop) or 2 signed oriented out-coming edges, hence giving at most two oriented circuits. The circuit direction is supposed to be imposed by the circuit nodes controlled by a boundary node: in the human genome, about 2,600 sources plus about 700 micro-RNAs (706 presently known after http://www.microrna.org and 800 expected [71]) are controlling 19,000 not boundary nor isolated genes [72], corresponding to one control among 4 not boundary nor isolated genes (if each micro-RNA is supposed to have on average three specific target genes), hence there is, on average, about 1 control per circuit of length 5, 4 or 3 (it is the case for the circuits in Figures 17 and 18).

**Figure 9.**
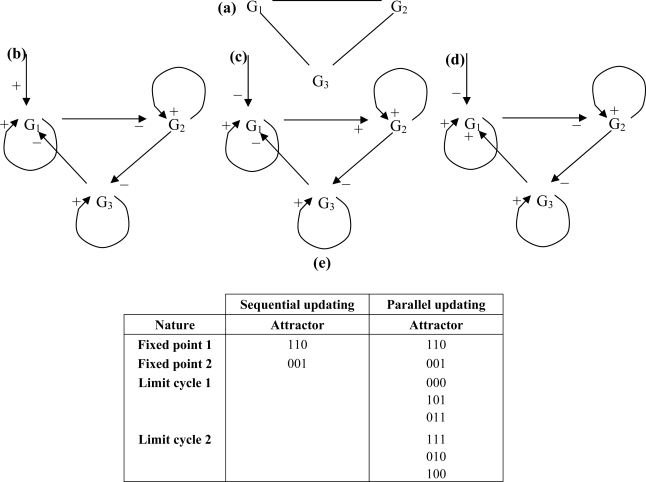
(a) undirected 3-circuit; (b) directed negative 3-circuit with positive control by a boundary gene; (c) directed positive 3-circuit with negative control by a micro-RNA or a boundary gene followed by a positive interaction; (d) directed positive 3-circuit with negative control by a micro-RNA or a boundary gene followed by a negative interaction; (e) attractors observed for the 3-circuit (c).

The circuit direction is chosen to give the priority to the first forward post-control interaction over the backward one. If the control is exerted on a 3-circuit, whose undirected graph is given in Figure 9 (a), each of its genes being auto-catalysed, and if we respect the graph constraints: c = 2; p_+_ = 4/7 (probability to have a sign +) and p_-_ = 3/7, the oriented graph is given in (b) if the control by a boundary gene is positive, and in (c) and (d) if the control by a micro-RNA or a boundary gene is negative (depending on the sign of the first post-control forward interaction).

### 4.6. Attractors Counting in Real Regulatory Networks

In 1949, M. Delbrück [8] conjectured that the presence of *positive circuits* (*i.e.*, paths from a gene i to itself having an even number of inhibitions) in the interaction graph of a genetic regulatory network was a necessary condition for the cell differentiation. This conjecture has been precisely written in a mathematical context by R. Thomas in 1981: he identified precisely in simple genetic regulatory networks each differentiated state with a specific attractor and he conjectured that the presence of at least one positive circuit in the interaction graph was a necessary condition for the existence of multiple fixed points [31].

In 1993, S. Kauffman [43] conjectured that the mean number of attractors for a Boolean genetic network with n genes and with connectivity *c* = 2, was of the same order of magnitude as *√(n)*. This conjecture was supported by real observations: there are about 22000 genes in the human genome and about 200 different tissues, which can be considered as different attractors of the same cell dynamics.

For *Arabidopsis thaliana*, the connectivity *c* is equal in the primitive graph to 25/12≈2 and there are 4 ≈ √(12) different tissues (sepals, petals, stamens, carpels) and for the Cro operon [21] of phage λ, *c* = 14/5 = 2.8 and there are *2* ≈ √(5) observed (lytic and lysogenic) attractors. Recently (cf. [32–40] and [71–78]), all these conjectures have been progressively made more precise and proven in specific contexts.

**Figure 10.**
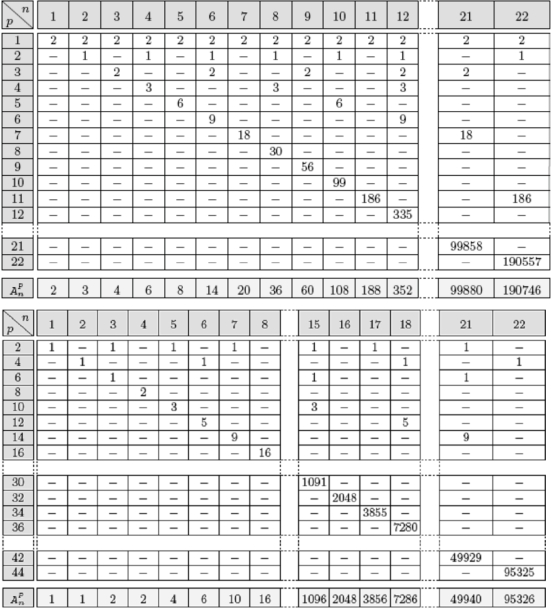
Top: Number of limit cycles of period *p* in parallel mode for a positive circuit of length *n*. Bottom: Number of limit cycles of period *p* for a negative circuit of length *n.* The last line of these tables gives the total number of attractor for circuits of size n.

Let us consider now directed random networks and try to evaluate the number of attractors brought by its circuits. By multiplying the numbers of attractors of the disjoint circuits, we can get a lower bound of the total number of attractors in the whole random network. For example, if *n* = 22.10*^3^*, then the mean number of oriented circuits of length *r* (for 3 *≤r ≤*5) is equal to *2^r−1^/r*≈ 16/5 ≈ 3, if *r* = 5, each bringing on average at most 6 different attractors in parallel updating mode [67]: 8 for positive and 4 for negative circuits (Figure 10) and 1.5 in sequential mode (2 fixed configurations for positive and 1 limit cycle for negative circuits). For *r* = 4 (resp. 3), the mean number of oriented circuits is equal to 2 (resp. 1) and the mean number of attractors is at most (6 + 2)/2 = 4 (resp. (4 + 2)/2 = 3). For *r* = 3, the attractors are given in Figure 9. For *r =* 2, the mean number of oriented circuits is equal to *n(*4*/n) =* 4, that is the number of the nodes of the network times the probability to have a reverse edge from the successor nodes of each node (whose mean number is equal to 2 in the case of connectivity 2). The number of attractors is 3 in the parallel mode of updating in the case of a positive circuit and 1 in the case of a negative circuit (Figure 10).

If the random oriented network contains *n* nodes and has a connectivity *c* (in the Kauffman’s sense) equal to 2 like in the *Arabidopsis* network [21] where *c* = 25/12 (Figure 7), then we can calculate the mean number *S* of sources; let us consider a network with *n* nodes and *2n* interactions; for each node, the probability of having one input from any of the *n* nodes interacting possibly with it (auto-interactions are allowed) and respecting the connectivity 2, is equal to 2/*n* ; after *n^2^* independent choices - *n* for each of the *n* nodes - the mean value of the total number of interactions is equal to *n^2^(*2*/n) =* 2*n*, that is the expected number of interactions; hence, the probability of having no input at is given by *p =* 1-2/*n*, and the probability of having no input for a node after *n* independent choices is *p^n^ = (*1-2/*n*)*^n^*. Then the mean number of sources is:

(11) $$S=n{\left(1-2/n\right)}^{n}$$

Because *(*1-2*/n)^n^* = *e^nLog(1-2/n)^* ≈ *e^−2^* ≈ 1/7.4, then *S* is approximatively equal to *n/*7.4, if *n* is sufficiently large. By using the same argument, the mean number of sinks *L* is equal to *S*, and the number *I* of isolated nodes is equal to about *n/(*7.4*)^2^* [81]. To conclude, the sources, if their *2^S^* states are possible, bring 2*^S^. A_n,2_* attractors, if *A_n,2_* is the number of attractors brought by the *n-(*2*S-I)* nodes having at least one in and one out interaction. These nodes belong to a random network having a connectivity (in the Kauffman’s sense) equal to *c′* = 2*n*/[*n-(*2*S-I)*] ≈ 2.8*n*. Then, for a fixed initial configuration of the sources, the calculation of attractors due to circuits inside these *n-(*2*S-I)* nodes can be made by using the previous Sections. The mean number of tree structures can also be calculated following [61], but these structures do not bring new attractors, except those brought by the sources and circuits connected to these trees.

These results are consistent with the fact that about 3,000 boundary genes, acting as sources, control the network of 22,000 genes of the human genome: the proportion of boundary genes is about 1/7 in some large regulatory networks such as the cell cycle control network [82], then the order of magnitude predicted in random digraphs is respected, if we are adding to the 22,000/7.4–22,000/(7.4)^2^ ≈ 2,973-402 = 2,571 sources or boundary genes, an amount of about 700 human micro-RNAs, which corresponds to the real size of the repositories (http://microrna.sanger.ac.uk; http://www.microrna.org) and to their expected size [79]. They control about 19,000 not boundary nor isolated genes [80], which corresponds to one control for every four not boundary nor isolated genes (each micro-RNA being supposed to have about three specific targets and hence to inhibit about 245 boundary genes), hence there is, on average, about 1 control per circuits of length 5, 4 or 3 (it is the case for the circuits in the Figures 17 and 18). A micro-RNA can hybridize a mRNA stopping the ribosomal elongation, then constituting a post-transcriptional control we can assimilate as coming from a new inhibitory source, *i.e.*, a boundary node acting negatively (rarely positively [81]) on the gene transcribed in this mRNA. The *n*-(G + L-I) nodes having at least one in and one out interaction are about 22,000-5,544 = 16,456 and they form about three circuits of length 5, 2 of length 4 and one of length 3. They bring 2^3^ attractors in the sequential mode and 8^1.5^.4^1.5^6^1^.2^1^.4^0.5^.2^0.5^ = 6.10^3^ attractors in the parallel mode. These numbers have been obtained by multiplying the number of the attractors brought by each positive or negative circuits, considered as disjoint and in equal average number.

If we consider that the boundary genes and micro-RNAs are always in state 1 of expression, the only possible changes in the attractors can come from an inhibition of the genes in state 1 of the positive circuits by the 700 micro-RNAs and the half of the 2,525 boundary genes non inhibited by the micro-RNAs: that corresponds to about 3,360 genes inhibited among the 19,000 not boundary nor isolated genes, that is 1/6 of these genes, *i.e.*, about 2 circuits of length 5, 1 circuit of length 4 and 0.5 of length 3. Then the control leaves free on average 1 circuit of length 5, 1 of length 4, 0.5 of length 3 and 1/3 of length 2 and there is on average in these free circuits at most 69 attractors in parallel updating, this number resulting from the calculation: 8^0.5^.4^0.5^.6^0.5^.2^0.5^.4^0.25^.2^0.25^.3^2/3^ = 3.6 × 2^4.25^ ≈ 69; this number 69 is in fact a majorant due to the Jensen’s inequality applied to the concave function *f(x)=x^p^*, where *p* < 1, the numbers of attractors, that is 8, 4, 6, 2 and 3, coming from the Figure 10 and the only non negligible circuits being of length less than 5, because of the Bollobas results [68]. The mean number of attractors allowed for the controlled circuits is in the same way equal at most to 8^0.5^.4^0.25^.6^0.25^≈ 4 × 1.56 ≈ 6.24, because the negative control acts efficiently only on genes in state 1, these genes concerning only the half of the positive controlled circuits. Then we can observe on average at most 69 × 6.24 ≈ 430 attractors, which is in qualitative agreement with the number of observed cell attractors in human (the number of the different types of cell differentiation in human is between 178 [82] and 411 [83]), if we suppose that the Boolean rules used in circuits of regulatory genetic networks are only made of identity and negation functions and that the genes are expressed synchronously (parallel updating). The pure sequential mode (8 attractors) is unable to explain the richness observed, hence we must propose a certain degree of synchrony in the gene expresssion, as well as new rules more sophisticated than the threshold Boolean automata one (*e.g.*, rules in which the interaction potential is non-linear in the state variables [61]).

## 5. Robustness

The robustness of a regulatory network is defined by its ability to resist to exogenous or endogenous perturbations, *e.g.*, to keep the same number and type of attractors (fixed or periodic), with about the same stability basin size. Exogenous perturbations from the environment can be caused by fluctuations (due for example to the nutrition) in the concentration of a protein active in the network (as repressor or inducer) or of a micro-RNA from viral origin acting on the mRNA transcribed from a gene of the network, or by a synchronisation of gene expression (due to chromosome rearrangements or to chromatin dynamics), provoking a change in attraction basins size or in type (nature or number) of attractors (*e.g.*, appearance of new limit cycles in Figure 7). An example of endogenous perturbation is a perturbation in the genetic co-expression scheme (due to the chromatin dynamics [84]) caused by a change in the updating rule of the network. For instance, the network may start with a sequential updating mode (all genes expressing their protein consecutively) which may turn into a block-sequential one (in each block the gene expression is synchronised) and eventually into the parallel updating mode (one sole block of genes all expressed simultaneously).

A threshold Boolean automata network rule can be implemented in 3 different updating protocols. Consider the example of a 3-switch (Figure 3 and 5), in which any gene inhibits the others with the weight −1 and is auto-catalysed with the weight + 1, the threshold being equal to -ε, where ε > 0:

- if the nodes are sequentially visited by the updating process, the system has 6 fixed configurations, with state 1 (resp. 0) at one node and state 0 (resp. 1) at the others. Such a system having only fixed configurations is potential in the sense of the Section 4.1. [64], because the discrete velocity of the dynamics is equal to the gradient of a Lyapunov function (it is for example more generally the case in a *n*-switch when the interaction weights are symmetrical),
- if the nodes are synchronously updated, we have one limit-cycle of order 2 (made of the full 0 and full 1 configurations) and 6 fixed configurations (corresponding to those of the sequential updating). Such a discrete system is Hamiltonian in the sense of Section 4.2.,
- in the intermediary case, called block-sequential, in which we update first a node, and then synchronously the two others, we have the same attractors as in the sequential case.

From this particular network of size 3, the 3-switch, we conjecture more generally that the cyclic behaviour can appear when the updating rule goes from the sequential to the parallel mode and not in the inverse way. The statistics done on the set of the all possible (188,968) networks of size 3 shows that this conjecture is almost everywhere true, but it fails for 0.3% of them [25,26,62]. Certain other empirical observations can be generalized:

**Lemma 2**: When a limit cycle occurs in the dynamics of a block, then the whole network dynamics has only limit cycles as attractors. Reciprocally, if the global network dynamics has a limit cycle, there is necessarily at least one block having at least one limit cycle as attractor.

**Lemma 3**: If the global network dynamics has a limit cycle of length *m*, then *m ≤ Π_i∈C_ m*_i_, where *C* is the set of the blocks having at least a limit cycle as attractor, *m_i_* being the maximal length of limit cycles of the block *i*.

The proofs of Lemmas 2 and 3 are obvious. The statistical behaviour of networks of size 3 needs a complete simulation of all their dynamics; we are only interested by networks of size 3 having some specific dynamical features: for certain updating modes, they have both fixed configurations and at least one limit cycle, the limit cycles disappearing completely for other iterations rules, where there are only fixed configurations. Among the 188,968 possible networks of size 3, corresponding to all the configurations of interactions and thresholds, only 34,947 (18.5%) have this dynamical behaviour. If we try to understand how limit cycles are disappearing when the iteration mode changes, frequently this disappearance occurs by passing from synchronous to sequential updating, in a hierarchy of updating modes, defined by the hierarchy of blocks based on the pre-order inclusion. We observe in the simulations three different possible behaviours for these 34947 size 3 networks:

- those for which the cycles disappear when we are going down in the hierarchy from the synchronous to the sequential modes (behaviour “Down”),
- those for which the cycles disappear when we are going up in the hierarchy from the sequential modes to the synchronous one (behaviour “Up”),
- those not corresponding to any previous behaviour, for which the cycles occur and disappear inside the hierarchy without clear rule (behaviour “None”).

Among the 34,947 simulated networks of size 3 having limit cycles, the dispatching into the 3 possible behaviours follows the repartition below.

This repartition confirms that there is practically no network (only 0.31%) for which the cycles are present in the sequential updating modes and disappear in the synchronous one.

Let us now consider the 108 networks with an “Up” behaviour, in order to try to find inside them a differentiation character. For that, let us calculate the maximal length of their limit cycles. For the 34,947 studied networks, this maximal length is equal to 6. The table below gives the repartition of the networks of size 3 as function of their maximal limit cycle length (the column “Total” corresponds to the repartition of the maximal lengths for the 34,947 simulated networks), showing the diversity in the response of networks of size 3 to the changes of the updating mode (cf. [27] for sizes from 4 to 7).

## 6. Examples of Robust and Non-Robust Networks

### 6.1. Neuron and Plant Morphogenesis

**Figure 12.**
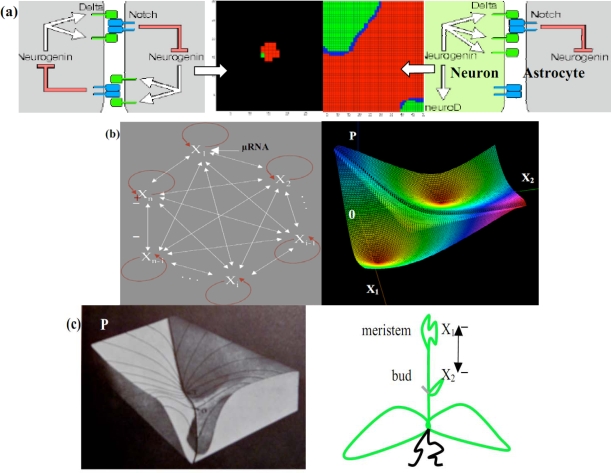
(a) Neuron/Astrocyte regulatory embryonic 2-switch (left). Co-evolution of the neuronal (red) and glial (green) tissue (middle). Neuron/Astrocyte adult network (right) (b) *n*-switch interaction graph (left). Potential *P* associated to a 2-switch (*ν =* 1, *c = σ =* 2, *a_i_ =.*1), with 2 stable minima on which vanishes either *X_1_* or *X_2_* (right). (c) Hard representation of the surface *P* (after [65]) (left). 2-switch plant growth (right).

**Figure 13.**
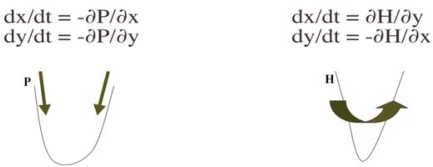
Potential (left) and Hamiltonian (right) systems.

Both brain and plants are built during their morphogenesis under the control of regulatory systems called *n*-switches [35,87], negatively fully connected, with an exception for positive auto-catalysis [Figure 12(b) left]. By considering a *n*-switch [88] available for the plant morphogenesis modeling [Figure 12(c) right], if *X_i_* denotes the concentration of the morphogen i, we suppose that all the inhibitions of the n-switch are expressed through a Hill competitive term in the following differential equations [83], where the cooperativity *c* is supposed to be strictly greater than 1, *k* is a catabolic constant and *σ*an enzymatic *Vmax* (Figure 13 left):

(12) $$dX_{i}/dt=-kX_{i}+\sigma X_{i}^{c}/(1+\sum_{j=1,n}X_{j}^{c}),\;\;\forall i=1,n$$

By doing the change of variables *Y_i_=(X_i_)^1/2^*, we can check that if we define the potential *P* by:

$$P(Y_{1,\ldots }Y_{n})=\sum_{j=1,\ldots ,n}kY_{j}^{2}/4-(\sigma /4n)\text{Log}(1+\sum_{j=1,\ldots ,n}Y_{j}^{2c}),$$

then new differential equations can be written as:

(13) $$dY_{i}/dt=-\partial P/\partial Y_{i},\;\forall i=1,\ldots ,n$$

The new differential system is then a gradient system with P as associated potential, whose minima are just located at the fixed points of the *n*-switch (Figure 12 b right and c left). This result is still available if the kinetics is allosteric, *i.e.*, if the inhibition is expressed through concerted sites in an oligomeric enzyme, that is, for every *i* between 1 and *n*:

$$dX_{i}/dt=-k_{i}X_{i}+\sigma X_{i}[{\left(1+X_{i}\right)}^{n-1}+Lc_{i}{\left(1+c_{i}X_{i}\right)}^{n-1}]/[{\left(1+X_{i}\right)}^{n}+L_{i}{\left(1+c_{i}X_{i}\right)}^{n}]$$

with *L_i_ = Π_k=1,n;k≠i_(1 + c_k_X_k_)^n^/(1 + e_k_X_k_)^n^* and *e_k_ << c_k_ <* 1, which corresponds to an allosteric inhibition of *X_i_* by the *X_k_*’s, for *k ≠ i*. If *e_k_ = 0*, by changing the variables *Y_i_ = (X_i_)*^1/2^ and considering the potential *P* defined by: $P(Y_{1},\ldots Y_{n})=\sum_{j=1,\ldots ,n}\;k_{j}Y_{j}^{2}/4-(\sigma /4n)\text{Log}(\sum_{j=1,\ldots ,n}\;{\left(1+Y_{j}^{2}\right)}^{n}+\prod_{j=1,\ldots ,n}\;{\left(1+c_{j}Y_{j}^{2}\right)}^{n})$, then the new differential equations can be written like (13) as:

(14) $$dY_{i}/dt=-\partial P/\partial Y_{i},\;\forall i=1,\ldots ,n$$

The applications of the *n*-switches concern the morphogenesis of the brain in which a 2-switch (made of transduction peptides in [89]) controls the interactions between early brain cells [Figure 12(a) left], the double inhibition leading to a spatial segregation between cell populations [Figure 12(a) middle] which differentiate in a second stage [Figure 12(a)right] giving for example separated (rods and cones in retina [90]) or intricate (neurons and astrocytes) complementary tissues. Many factors that control cells fate are themselves targets of such inhibitory proteins: neural induction in vertebrates [91,92] as well as plant growth [88] with inhibition between meristem and buds may be mediated through such a mechanism [Figure 12(c) right] very sensitive to the initial conditions; each attractor corresponds to the local dominance of a node of the *n*-switch over the others [Figure 12(b) left].

### 6.2. Cardio-Respiratory Physiologic Regulation

**Figure 14.**
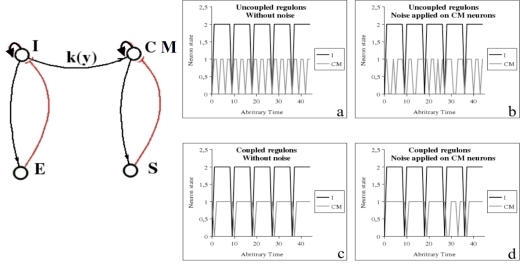
Left: Bulbar cardio-respiratory centre, with inspiratory *I* and expiratory *E* neurons, cardio-moderator *CM*, peripheral sinusal pace-maker S. Right: (a) Periodic dynamics of *I* and *CM* (3-harmonic signal) when they are uncoupled or weakly coupled without noise, (b) respiratory sinusal arrhythmia with noise, (c) pathological entrainment when they are strongly coupled without noise and (d) with noise.

The bulbar vegetative regulation of the cardio-respiratory system is made of three main neuronal populations [93], firstly the inspiratory neurons *I* activating the expiratory neurons *E*, with an inhibitory feed-back on *I*, and secondly the cardio-moderator *CM* activating the peripheral cardiac pace-maker made of the excitable cells of the sinusal node *S*, with an inhibitory feed-back on *CM* (Figure 14 left). In the healthy state, the cardiac activity is ruled by these two negative regulons and in presence of a weak coupling between *I* and *C*, the cardiac rhythm is just the 3-harmonic component of the respiratory one (Figure 14 right a), presenting an acceleration during inspiration, called respiratory sinusal arrhythmia, visible in presence of noise (Figure 14 right b), and destroyed in case of degenerative neural disease (like in Parkinson’s or Alzheimer’s disease, where there is no more coupling). A too strong coupling between *I* and *CM* transforms the 3-harmonic cardiac rhythm in a pathological signal having same frequency as the respiration (Figure 14 right c and d). The presence of a negative regulon causes the occurrence of an attractor, which is a limit cycle, as usually [93–95].

### 6.3. Glycolytic/Oxidative Coupling

#### 6.3.1. The Glycolysis

**Figure 15.**
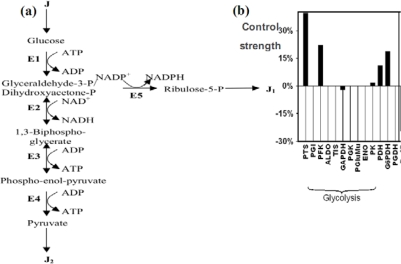
(a) The glycolysis and the pentose pathway. E1 denotes the four enzymes of the high glycolysis (hexokinase HK, phosphoglucose-isomerase PGI, phosphofructo-kinase PFK and aldolase ALDO), E2 denotes the glyceraldehyde-3P-dehydrogenase, E3 denotes the four enzymes of low glycolysis (phosphoglycerate-kinase, phosphoglycerate-mutase, enolase ENO and pyruvate-kinase PK), E4 denotes the pyruvate-kinase and E5 the 3 enzymes of the oxydative part of pentose pathway, glucose-6P-dehydrogenase G6PDH, 6P-glucono-lactonase and phosphogluconate-dehydrogenase PGDH, alternative to the phospho-transferase system PTS). (b) Control strengths on the glucose flux showing the influence of the main enzymatic steps.

The glycolysis [Figure 15(a)] has been modeled multiple times by several authors [96–102], especially the central allosteric step of the phosphofructokinase (PFK), which is the key glycolytic/oxidative enzyme, because it presents a highly non-linear allosteric kinetics (with cooperativity equal to six) and has an ATP/ADP negative regulon in its regulatory interaction network, causing, for critical values of the constant fructose entry flow σ_1_, oscillations of all the metabolites of the glycolysis, with a period of several minutes. Let us apply it to neurons and astrocytes: the mitochondrial shuttles for NADH are less active in astrocytes than in neurons: that causes a flow of lactate from astrocytes to neurons [101,102] and this difference in NADH transportation efficiency is provoked by a weaker efficacy of the translocase (ANT) and ATPase enzymes less optimally located inside the inner mitochondrial membrane [103].

Let us define now by *x*_1_*, x_2_, x_3_* and *x_4_* the concentrations of respectively the successive main metabolites of the glycolysis: glucose, glyceraldehyde-3-P, 1,3-biphospho-glycerate and phospho-enol-pyruvate. We assume that steps E2 and E3 of the glycolysis (summarised in Figure 15 a) are Michaelian and reversible, the enzymatic complex E1 includes the allosteric irreversible kinetics of the phospho-fructo-kinase PFK with a cooperativity *n* (see [104–108] for its complex kinetics), and both pyruvatekinase (E4) and dehydrogenases of the complex E5 are irreversible. Then, consider the differential system (S) ruling the glycolysis and the pentose pathway until the ribulose-5-P:

$$\begin{array}{c} dx_{1}/dt=J-V_{1}x_{1}^{n}/(1+x_{1}^{n}),\;dx_{2}/dt=V_{1}x_{1}^{n}/(1+x_{1}^{n})-V_{2}x_{2}/(1+x_{2})+L\alpha V_{2}x_{3}/(1+x_{3})\\ dx_{3}/dt=\alpha V_{2}x_{2}/(1+x_{2})-V_{3}x_{3}/(1+x_{3})-L\alpha V_{2}x_{3}/(1+x_{3})+L^{\prime }V_{3}x_{4}/(1+x_{4}),\\ dx_{4}/dt=V_{3}x_{3}/(1+x_{3})-V_{4}x_{4}/(1+x_{4})-L^{\prime }V_{3}x_{4}/(1+x_{4}) \end{array}$$

Let us consider the change of variables: $y_{i}=x_{i}^{1/2},\;dy_{i}/dx_{i}=x_{i}^{-1/2}/2$. The *y_i_*’s are ruled by the system (S′):

$$\begin{array}{c} dy_{1}/dt=[J-V_{1}y_{1}^{2n}/(1+y_{1}^{2n})]/2y_{1},\;dy_{2}/dt=[V_{1}y_{1}^{2n}/(1+y_{1}^{2n})-V_{2}y_{2}^{2}/(1+y_{2}^{2})+L\alpha V_{2}y_{3}^{2}/(1+y_{3}^{2})]/2y_{2}^{,}\\ dy_{3}/dt=[\alpha V_{2}y_{2}^{2}/(1+y_{2}^{2})-V_{3}y_{3}^{2}/(1+y_{3}^{2})-L\alpha V_{2}y_{3}^{2}/(1+y_{3}^{2})+L^{\prime }V_{3}y_{4}^{2}/(1+y_{4}^{2})+]/2y_{3},\\ dy_{4}/dt=[V_{3}y_{3}^{2}/(1+y_{3}^{2})-V_{4}y_{4}^{2}/(1+y_{4}^{2})-L^{\prime }V_{3}y_{4}^{2}/(1+y_{4}^{2})]/2y_{4} \end{array}$$

Let us consider now the potential *P* and the energies *H_i_* [109] defined by:

$$\begin{array}{c} P=-J\text{Log}(y_{1})/2+V_{1}\text{Log}(1+y_{1}^{2n})/4n+V_{2}\text{Log}(1+y_{2}^{2})/4+(V_{3}+L\alpha V_{2})\text{Log}(1+y_{3}^{2})/4+(V_{4}+L^{\prime }V_{3})\text{Log}(1+y_{4}^{2})/4\\ H_{1}=-V_{1}\text{Log}(1+y_{1}^{2n})/8ny_{2}+L\alpha V_{2}\text{Log}(1+y_{3}^{2})/8y_{2},\;H_{2}=-\alpha V_{2}\text{Log}(1+y_{2}^{2})/8y_{3}+L^{\prime }V_{3}\text{Log}(1+y_{4}^{2})/8y_{3},\\ H_{3}=-V_{3}\text{Log}(1+y_{3}^{2})/8y_{4} \end{array}$$

Then, we have:

$$\begin{array}{c} dy_{1}/dt=-\partial P/\partial y_{1},dy_{2}/dt=-\partial P/\partial y_{2}-\partial H_{1}/\partial y_{1}+\partial H_{1}/\partial y_{3},dy_{3}/dt=-\partial P/\partial y_{3}-\partial H_{2}/\partial y_{2}+\partial H_{2}/\partial y_{4},\\ dy_{4}/dt=-\partial P/\partial y_{4}-\partial H_{3}/\partial y_{3}, \end{array}$$

and, when the variables *y_i_*’s are large, the gradient of *P* dominates and the system has a principal potential part [110], this part giving then the direction of the flow. Conversely, when the gradient of *P* vanishes, the part made of the partial derivatives of the *H*_i_’s dominates.

#### 6.3.2. Control Strength

Let us share the velocity field *dy/dt*, where *y = {y_i_}_i=1,4_*, between two parts, one gradient part dissipating the potential *P* and the other part made of the partial derivatives of the *H_i_*’s ; that allows, when the attractor is a limit cycle - which is the case if we add fructose-2,6-diphosphate (F26P_2_) or ADP (see Figures 11 a, 13 left and 14 right) as activator of the complex E_1_ [99,108] - to share the parameters of the system (S’) into 2 sets: the parameters appearing exclusively in *P* modulating the mean value and the amplitude, the parameters appearing exclusively in the *H_i_*’s modulating more the frequency and those appearing both in *P* and in the *H_i_*’s.

When the attractor is a fixed point, the first enzymatic complex E1 has a stable stationary state defined by $V_{1}x{*}_{1}^{n}/(1+x{*}_{1}^{n})=J$. If this value x*_1_ is reached the first among the other x*_k_’s, then (S’) becomes (S″):

$$\begin{array}{c} dy_{2}/dt=J-\partial P*/\partial y_{2}+\partial H_{1}*/\partial y_{3},dy_{3}/dt=-\partial P*/\partial y_{3}-\partial H_{2}*/\partial y_{2}+\partial H_{2}*/\partial y_{4},\;\;dy_{4}/dt=-\partial P*/\partial y_{4}-\partial H_{3}*/\partial y_{3},\;\text{where}:\\ P*=-J\text{Log}(y_{2})/2+V_{2}\text{Log}(1+y_{2}^{2})/4+(V_{3}+L\alpha V_{2})\text{Log}(1+y_{\;3}^{2})/4+(V_{4^{-}}+L^{\prime }V_{3})\text{Log}(1+y_{4}^{2})/4,\\ H{*}_{1}=Jy_{3}/2y_{2}+L\alpha V_{2}\text{Log}(1+y_{3}^{2})/8y_{3},\;H{*}_{2}=\alpha V_{2}\text{Log}(1+y_{2}^{2})/8y_{3}+L^{\prime }V_{3}\text{Log}(1+y_{4}^{2})/8y_{3},\\ H{*}_{3}=-V_{3}\text{Log}(1+y_{3}^{2})/8y_{4} \end{array}$$

In this case, parameters appearing only in P*, like *V_4_*, are modulating the localisation of the fixed point, hence the values of the stationary concentrations of the glycolytic metabolites (cf. [109] for a more general approach of the potential-Hamiltonian decomposition).

Let us suppose now that we measure the outflows *J_1_* and J_2_. Then from the system (S) we can calculate the sharing parameter α (which regulates the pentose pathway and the low glycolysis dispatching) from the steady-state equations equalizing the in and outflows at each step. By determining the stationary state x̄* = {x_i_}_i=1,4_, we have:

$$\begin{array}{c} V_{1}x{*}_{1}^{n}/(1+x{*}_{1}^{n})=J,\;V_{2}x{*}_{2}/(1+x{*}_{2})-L\alpha V_{2}x{*}_{3}/(1+x{*}_{3})=J,\;(1-\alpha )V_{2}x{*}_{2}/(1+x{*}_{2})=J_{1}^{,}\\ V_{3}x{*}_{3}/(1+x{*}_{3})=J_{2}(V_{4}+L^{\prime }V_{3})/V_{4} \end{array}$$

Hence, we can calculate *α*, the repartition coefficient between the low glycolysis and the pentoses pathway, by using the following formula:

$$\begin{array}{c} \alpha^{2}J_{2}LV_{2}(V_{4}+L^{\prime }V_{3})+\alpha (JV_{4}V_{3}-J_{2}LV_{2}(V_{4}+L^{\prime }V_{3}))+J_{1}V_{4}V_{3}=0\;or\;\alpha^{2}-\alpha (1-K)+K^{\prime }=0,\\ \text{where}\;K=JV_{4}V_{3}/J_{2}LV_{2}(V_{4}+L^{\prime }V_{3}))\;and\;K^{\prime }=J_{1}V_{4}V_{3}/J_{2}LV_{2}(V_{4}+L^{\prime }V_{3})) \end{array}$$

When all the fluxes of a metabolic system have reached their stable stationary value, then we can define the notion of control strength *C_ik_* exerted by the metabolite *x_i_* on the flux *Φ_k_* of the k^th^ step by: *C_ik_* = *∂LogΔΦ_k_/∂LogΔx_i_* [105,111,112] and we have:

(15) $$\forall k=1,n,\;\;\sum_{i=1,n}\;C_{ik}=1$$

The control molecules can be enzymes [Figure 11(b)] or metabolites and the equation (14) can be used to prove that the most regulating molecules in glycolysis are the energetic molecules ATP and ADP, which conversely are mainly produced by glycolysis: for example, 84% of the ATP production in yeast is provided and controlled by glycolysis [113].

When oscillations occur, we can use the variables *T_ik_* (resp. *A_ik_*) to quantify the control of the period *τ_k_* (resp. amplitude *Amp_k_*) of the k^th^ step flux by *x_i_* [112–114]: *T_ik_=∂Logτ_k_/∂Logx_i_* and *A_ik_ = ∂LogAmp_k_/∂Log*x_i_. If *ξ* is the eigenvalue of the Jacobian matrix of the differential system for which the stationary state has bifurcated in a limit cycle (Hopf bifurcation), then *τ_k_ =* 2*π/Imξ*, and we have in the 2-dimensional case, if for example *dx_1_/d t= −∂P/∂x_1_+∂H/∂x_2_, dx_2_/dt=-∂P/∂x_2_-∂H/∂x_1_*:

$$Im\;\xi =/{\left(\Delta P\right)}^{2}-4(C(P)+C(H)+\partial^{2}P/\partial x_{1}\partial x_{2}(\partial^{2}H/\partial x_{2}^{2}-\partial^{2}H/\partial x_{1}^{2})+\partial^{2}H/\partial x_{1}\partial x_{2}(\partial^{2}P/\partial x_{1}^{2}-\partial^{2}P/\partial x_{2}^{2}){/}^{1/2}/2,$$

where *ΔP = ∂^2^P/∂x_1_^2^*+ *∂^2^P/∂x_2_^2^* is the Laplacian of *P* and *C(P) = ∂^2^P/∂_1_^2^∂^2^P/∂x_2_^2^*- (*∂^2^ P/∂x_1_ ∂x_2_*)*^2^* is the mean Gaussian curvature of the surface *P*, both taken at the stationary state of the differential system.

**Figure 16.**
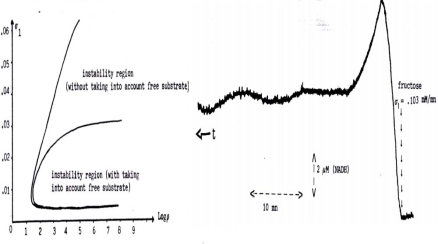
Left. Parametric instability region reduced by taking into account the fraction of metabolites fixed to PFK when its *V_max_* is large, entry flux of fructose *σ_1_* is small and ratio *ρ = K_R,F6P_/K_R,ADP_* between association constants of Fructose-6-Phosphate and ADP to the active allosteric form R of the PFK is large. Right. Experimental evidence of oscillations in the large parametric instability region (*V_max_* large) [99].

**Figure 17.**
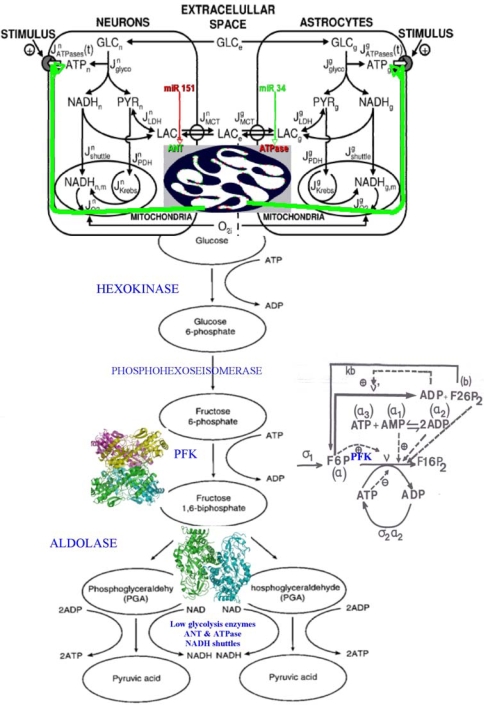
Left. Connection between neurons and astrocytes glycolysis with lactate flux, controlled by ANT (green) and ATPase (red) inside the mitochondrial inner membrane (top). Right. PFK regulatory interaction network with the effectors (activators and inhibitors) of the PFK inside the enzymatic complex E_1_.

When the apparent *V_max_* of the PFK is diminished in astrocytes due to a lack of ATP, the oscillatory behaviour is less frequent (Figure 16 left) and the production rate of lactate from pyruvate is more important than in neuron, creating a flux of lactate to neurons (Figure 17 top). The neurons consume the lactate coming from the extracellular space, partially replenished by the astrocytes production. That gives to neurons an ATP level higher than in astrocytes, an extra-pyruvate production from lactate, and an extra-oxygen and glucose consumption theoretically predicted and experimentally observed [101,102]. ANT and ATPase concentrations are in human under the negative control of two micro-RNAs, micro-RNA 151 and micro-RNA 34 acting as boundary controller nodes (http://microrna.sanger.ac.uk) by diminishing the glycolytic/oxidative system efficiency.

### 6.4. Cell Cycle Control

**Figure 18.**
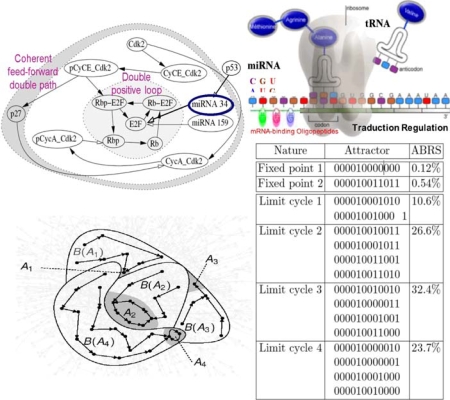
Cell cycle network (top left). Micro-RNA traductional regulation (top right). Some attractors (bottom left) in an arbitrary state space and list of all attractors of the cell cycle network, with fixed boundary conditions (micro-RNAs in state 1) and parallel updating mode (bottom right). ABRS denotes the percentage of initial states in an attractor basin. The involved genes are: p27, Cdk2, pCyCE_Cdk2, CyCE_Cdk2, micro-RNA 159, pCycA_Cdk2, CycA_Cdk2, Rbp-E2F, Rb-E2F, E2F, Rbp and Rb. Arbitrary representation of a part of the attraction basins with only 2 fixed points, A_1_ and A_3_, and 2 limit cycles, A_2_ and A_4_ (bottom left).

The cell cycle is sensitive to the control by two micro-RNAs whose one (micro-RNA 34) has p53 as positive transcription factor, involved in selective cytotoxicity of intracellular amyloid [115–117]. Fixing to state 1 micro-RNAs causes the occurrence of limit cycles in the parallel mode (Figure 18).

### 6.5. Feather Morphogenesis

**Figure 19.**
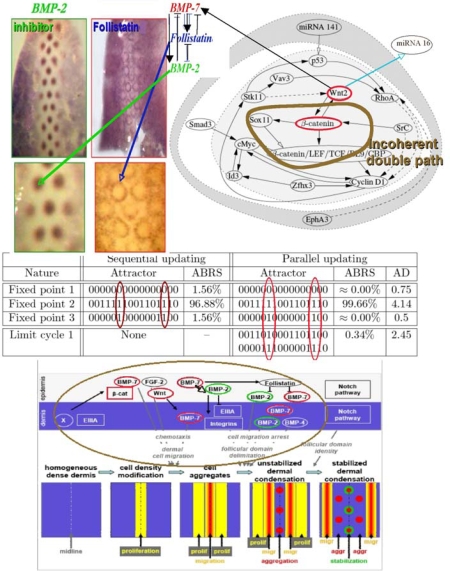
Feather morphogenesis network (top right) connected to a double negative regulon with BMP-2 as inhibitor, BMP-7 as activator, and Follistatin as intermediary controller identified by specific dyes on the back of chicken ambryo (top left). Simulated attractors, where AD denotes the attractor basin diameter in Hamming distance with nodes in the order: miRNA 141, EphA3, p53, Vav3, Stk11, Wnt2, RhoA, Smad3, SrC, Id3, Cyclin D1, Zfhx3, Sox11, *β*-catenin, cMyc, and *β*-catenin/LEF/TCF/BL9/CBP (middle). Morphogenetic targets of the regulatory network (bottom).

The feather morphogenesis network has been well studied in chicken [118–125] and is described in Figure 19. One boundary node, the micro-RNA 141 inhibits the protein p53 involved in the positive control of the Cyclin D1 and in the negative control of E2F via the micro-RNA 34, whose p53 is a transcription factor (Figure 18 top left). Hence the micro-RNA 141 can have a double action on the proliferation (modeled by using the Ross-Volterra logistic term [126,127]), by breaking the p53 influence. If the boundary state of micro-RNA 141 is equal to 1, we notice a dramatic change in the number and nature of attractors, because we lose both in the parallel and sequential cases one fixed attractor by changing the state of micro-RNA 141 from 0 to 1 and we transform the limit cycle of the parallel updating case in a fixed point. In these new attractors, there are still two types of behaviour, one with Wint (Wnt) and β-catenin silenced causing the absence of the activator BMP-7, hence the absence of feathers, and another with Wint and β-catenin expressed, allowing the feather morphogenesis. Hence, the node micro-RNA 141 is critical and the feather morphogenesis network is sensitive to its influence, but anyway even in its absence the possibility to start or not the feather morphogenesis by silencing or enhancing Wint and β-catenin remains possible, *e.g.*, through the gene Smad3 (Figure 19 top right).

## 7. Perspectives and Conclusions

We have studied in this paper the robustness of regulatory interaction networks, especially the influence of boundaries in threshold Boolean automata networks. The sensitivity (or absence of robustness) in real biological networks appears dominant in the case of inhibitory actions exerted by micro-RNAs and we have noticed it in both cases of the cell cycle control and the feather morphogenesis regulation networks. We have also remarked the dependence on the updating mode [128] in the simulations and the role of positive circuits on the number and nature of attractors (especially in the *Arabidopsis* flowering network and in the *n*-switches), the influence of negative circuits (*e.g.*, coming from simple negative regulons) on the existence of periodic behaviours (namely in the cases of the cardio-respiratory control network and of the glycolytic-oxidative coupling). More systematic studies as in [129,130] have to be performed in order to confirm the dominant influence of boundary negative interactions, for which the threshold Boolean automata networks seem to be less robust than for the positive ones, and also to make more precise the conjectured inequality about the number of attractors (verified here in each discrete threshold Boolean automata network studied in the examples given here as application of the systems biology approach). Many problems remain open: for instance, we could consider the regulatory networks in an evolutionary perspective, in order to determine phylogenetic trees of networks (by using classical distances between networks, like the tree distances [131–136]). We could indeed show that the known regulatory networks have evolved following a complexification such as the number of nodes at step *k, n(k)*, verifies: *n(k) = C.*2*^Log(r(k))Log(Log(r(k)))^*, where *C* is a positive constant and the directed graph diameter is equal to 2*r(k)* at step *k*. Then, if the indegree of the interaction graph comes from the initial value 2, it loses this indegree not too rapidly and goes to a small-world structure [137], like those observed in the metabolic regulatory networks [1].

More, let us consider the notion of r-*tree*, *i.e.*, an undirected connected graph without circuits, but with a root and with each vertex of any level *i* from this root (except sinks) having *r* adjacent vertices at level *(i +* 1*)*. If the number *m(k)* of the edges of the undirected graph associated to a random regulatory network at step *k* verifies:

$$m(k)=O{\left(K\;n(k\right)}^{(r-2)/r}[n(k)-1]/2)$$

where *K* and *r* are positive constants, then the number of r-trees of size *r +* 1 is on average equal to *K^r(r+1)/2^/(r +* 1*)!* [68,138–142]. All these trees controlled by their root bring each only one attractor. Following [143], if we consider for example the network of the copper biolixiviation by *Thiobacillus ferrooxidans* [144,145], we have 354 genes and 534 interactions: the prediction for the numbers of isolated genes and 2-trees are 354/(7.4)^2^ = 6.5 and 3^3^/3! = 4.5, and the observations show 8 isolated genes and 6 2-trees, in qualitative agreement with the hypothesis of randomness. This result emphasizes the role of circuits in the differentiation, because positive ones are alone to bring multiple fixed points and be present since the origin of life [131]. A last open problem consists in making explicit the dynamical consequences of the transcription regulation of micro-RNAs, by boundary genes like p53 [117]: this regulation enlarges the classical boundary reduced to sources, by introducing 2-circuits in it.

To conclude, all the predictions from the theoretical models have to be falsifiable by the empirical observations, reinforcing the interest of the methodological approach, summarised here shortly in the framework of the systems biology by using the tools of the dynamical systems and complex systems theories, and applied in various biological fields, from genetic and metabolic to physiologic control.

## Figures and Tables

**Table 1.** Repartition of the 34,947 networks of size three having limit cycles into three classes: Down (resp. Up) for which cycles disappear when the updating mode loses its synchrony (resp. sequentiality) and None for which cycles occur and disappear without clear rule.

**Down**	**None**	**Up**	**Total**

21,729	13,110	108	34,947
62.18%	37.51%	0.31%	100%

**Table 2.** Repartition of the 34947 networks of size 3 having limit cycles of length 2 to 6 into the 3 classes Down, None and Up.

	**Down**	**None**	**Up**	**Total**

**2**	86.19%	70.53%	37.04%	80.16%
**3**	8.28%	20.93%	62.96%	13.20%
**4**	4.59%	6.71%	0.00%	5.37%
**5**	0.70%	1.83%	0.00%	1.12%
**6**	0.24%	0.00%	0.00%	0.15%
